# Relationship between Swimming Performance, Biomechanical Variables and the Calculated Predicted 1-RM Push-up in Competitive Swimmers

**DOI:** 10.3390/ijerph182111395

**Published:** 2021-10-29

**Authors:** Sofiene Amara, Oussama Gaied Chortane, Yassine Negra, Raouf Hammami, Riadh Khalifa, Sabri Gaied Chortane, Roland van den Tillaar

**Affiliations:** 1 Higher Institute of Sport and Physical Education of Ksar-Said, University of La Manouba, Tunis 2010, Tunisia; oussama.gaeid@gmail.com (O.G.C.); yassinenegra@hotmail.fr (Y.N.); raouf.cnmss@gmail.com (R.H.); riadhkhal@yahoo.fr (R.K.); 2 Research Unit (UR17JS01) Sports Performance, Health & Society, Higher Institute of Sport and Physical Education of Ksar-Said, Universite de la Manouba, Tunis 2010, Tunisia; 3 Laboratory of Cardio-Circulatory, Respiratory, Metabolic and Hormonal Adaptations to Muscular Exercise, Faculty of Medicine Ibn El Jazzar, Sousse 4002, Tunisia; Sabrigaied1@gmail.com; 4 Department of Sport Sciences and Physical Education, Nord University, 8026 Levanger, Norway

**Keywords:** strength, stroke length, stroke rate, upper body

## Abstract

One repetition maximum (1RM) push-ups, based upon the load–velocity relationship, are able to predict the maximum upper body strength. The aim of the present study was to examine the relationship between the predicted 1RM push-up based upon the load–velocity relationship and swimming performance and kinematical variables in competitive swimmers. Thirty-three competitive male swimmers (age = 16.46 ± 0.59 years, body mass = 72.82 ± 8.41 kg, body height = 180.56 ± 5.69 cm) performed push-up exercises without a weight vest and with a 10, 20 and 30 kg weight vests. A load–velocity relationship was established as a product of the load and velocity of the push-up per participant, and the equation was used to establish a predicted 1RM. Our findings showed a predicted 1RM push-up of 82.98 ± 9.95 kg. Pearson correlations revealed a nearly perfect relationship between the 1RM push-up and the 25 or 50 m front crawl (r = −0.968, r = −0.955), and between 1RM push-up and the 25 or 50 m front crawl with arms (r = −0.955, r = x0.941). Similarly, our results revealed significant near-perfect correlations between 1RM push-up and kinematical variables (r = 0.93–0.96) except the stroke index, which had a large relationship (r = 0.56). This study suggests that swimming performance and kinematical variables are correlated with the predicted 1RM push-up. The 1RM push-up based upon the load–velocity relationship is a low cost and time-effective alternative for swimmers and coaches to predict maximum upper body strength to optimize swimming performance in short races.

## 1. Introduction

Swimming is a big aquatic sport, with different disciplines, of which the crawl is one. Optimizing front crawl performance requires improving kinematical and physical abilities [1,2]. One of those abilities in front crawl performance is related to the upper body strength [3,4]. Specifically, maximum upper and lower body strength exhibit a strong correlation (r = 0.67–0.79) with swimming performance over less than 100 m [4].

Therefore, several swimming studies have conducted muscle strength training, aiming to improve the maximum upper and lower body strength in relation to increased swimming performance. Several resistance exercises have been performed, such as the bench press (BP), medicine ball throw (MBT), back squat (BS), and leg extension (LE) with different intensities and loads [1,5,6]. In the same context, Lopes et al. [1] revealed that 8 weeks of dry-land training including BP and MBT exercises improved the upper body strength (one repetition maximum [1RM] bench press: 12.98%), kinematical variables (stroke rate in 50 m: 12.20% and in stroke index in 100 m: 5.86%), and swimming performance (50 m: 3.98% and 100 m: 4.34%) of competitive sprinter swimmers.

There are different ways to measure upper body strength. For instance, bench press [1,4] and isokinetic dynamometer [5,6] or a Technogym cable crossover apparatus [7] are exercises frequently used as measurements of maximum upper body strength. However, these methods are generally performed in laboratories and weight rooms, and some measurements are very time-consuming or need equipment.

The push-up exercise is a possible exercise that can also measure upper body strength instead of the bench press [8]. Van den Tillaar and Ball [8] showed a nearly perfect correlation between the predicted 1RM push-up and 1RM bench press based upon the load velocity relationship with a submaximal load (ICC = 0.96, r = 0.93) in resistance-trained male athletes. While the 1RM bench press exercise is a good predictor of performance in the 50 and 100 m front crawl [4] it is not known if this push-up exercise has a good relationship with the kinematical variables and swimming performance.

Therefore, the aim of the present study is to investigate whether the predicted 1RM push-up, based upon the load–velocity relationship of a submaximal load, has a strong relationship with 25 and 50 m front crawl swimming performance and their kinematical variables (velocity, stroke rate, stroke length, and stroke index) in competitive swimmers. Based upon the study by Keiner et al. [4] that showed a strong correlation between bench press performance and swimming performance, and the study of van den Tillaar and Ball. [8] on similarities between the bench press and push-ups, we hypothesize that there is a very strong correlation between push-ups and swimming performance. If there is a very strong relationship, this could be an easier and cheaper method to help coaches and swimmers evaluate upper body strength in relation to swimming performance.

## 2. Materials and Methods

### 2.1. Experimental Approach to the Problem

To investigate the relationship between maximum upper body strength determined by the predicted 1-RM push-up and swimming performance variables, a load–velocity relationship was established by evaluating the velocity at four different sub-maximum loads in push-ups. The 1RM push-up was predicted based on the load–velocity relationship established by a prediction equation. The predicted 1-RM push-up load was used as the independent variable, while the swimming performance (25 and 50 m front crawl, and 25 and 50 m front crawl with arms only) and kinematical variables of the 50 m front crawl (velocity, stroke length, stroke rate, and stroke index) were the dependent variables. The present study was performed during the competitive season (February–April).

### 2.2. Participants

Thirty-three national competitive male swimmers (age = 16.46 ± 0.59 years, body mass = 72.82 ± 8.41 kg, body height = 180.56 ± 5.69 cm) with previous dry land resistance training experience (6.08 ± 0.37 years) and swimming training experience (9.50 ± 0.71 years) volunteered to participate in the study. Swimmers performed approximately six water-training sessions per week (between 4000 and 6000 m per session) and two dryland sessions (general strength training) before the present study. The swimmers were sprinters (mean personal best performance in 50 m front crawl was 25.01 s). Participants were asked to avoid any additional resistance training targeting the upper body for 72 h before the test. Written consent was obtained from each swimmer and their guardians prior to the study. This investigation complied with the ethical regulations in force for research and was approved by the ethics committee of Higher Institute of Sport and Physical Education of Ksar Said, University of Manouba, Tunisia (Research Unit UR17JS01, Sports Performance, Health and Society) and in line with the underlying the latest Declaration of Helsinki of 2013. All physical experience and anthropometric characteristics are presented in Table 1.

### 2.3. Procedures

#### 2.3.1. Maximum Strength Prediction Test

**Figure 1:**
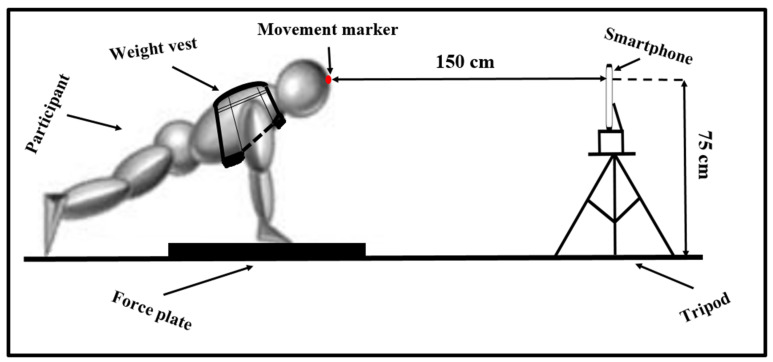
Push-up test condition performed by the participant and the setting up of test equipment.

The swimmers visited the laboratory (22.3 °C air temperature, 45% relative humidity) twice, separated by 48 h, at the same time, on two days (10.00 AM) and all participants were informed to eat their breakfast minimum 3 h before the start of the test, and to avoid all other nutrition, including caffeine or other nutrition. The first time was for a familiarization session. The second visit was for a test session, which started with the anthropometric measurements of the swimmers (height and body mass). Subsequently, each subject performed a standardized warm-up consisting of a 5 min run on a treadmill at a sub-maximal level (8–10 km/h). After the warm-up, participants performed the push-up test with four different loads: own body mass and with a 10, 20, and 30 kg weight vests (Titan Fitness, Memphis, TN, USA). Three repetitions per load were performed, and the different loads were given in ascending or descending order, equally randomized for each subject [8]. Rest intervals of 3 to 5 min were allowed between each load to avoid fatigue. Participants used their preferred grip width in the push-up, and this was measured and normalized for each load maintained during the exercise. The push-up depth was qualitatively validated by the main height of each repetition. To avoid risk of injury, no actual 1RM testing was performed in this study. The push handles were placed on a force plate (9290AD; Kistler, Winterthur, Switzerland), which sampled at 500 Hz. The participant’s feet were placed behind the plate at the same height as the force plate (Figure 1). The initial ground reaction force was measured when the participant was in the starting position and bearing their full weight on the lift handles. The reaction force of the ground at the starting position was then used to calculate the absolute load and the percentage of body weight (without and with weight vest) to be lifted during the different push-up conditions [8]. The test was controlled by two strength and conditioning coaches.

A smartphone (Redmi Note 8, Xiaomi, Beijing, China) with a high-speed camera (Snapdragon 886 processor at 2.0 GHz and 48 + 8 + 2-megapixel quad-cameras) was used to film the push-up exercises. All videos were recorded in the slow-motion option at 240 fps in automatic mode. The smartphone was attached to a tripod 150 cm from the focal plane to the plane of the movement marker placed on the participant’s head. In addition, the main lens of the camera was placed at a 75 cm height [9]. The video recordings of the push-ups were processed with Kinovea software, version 0.8.15 (Joan Charmant & Contrib., kinovea.org, accessed on 25 October 2021) [10] with a frequency of 240 Hz to ensure the correct calculation of the execution speed [11,12,13]. A point marker was put on the head of each swimmer. In addition, the displacement of the point maker during the descending and ascending phases of the push-up was limited by two imaginary lines added to the 2D plane in the Kinovea software. The mean concentric velocity was calculated from the distance between the lowest position and the highest position according to the imaginary digital lines, and time it took to cover this distance.

The load–velocity relationship was established for the push-up exercise by the average velocity of the three repetitions of the four different loads performed by each participant, as determined by the Kinovea software. Based on the athlete’s performance at different loads, linear regression was used to predict the 1RM push-up for each participant.

To calculate the predicted 1RM push-up, the following formula was used:

y = a × x + b(1)

The variable x was set at 0.18 m/s, which indicated the minimum mean propulsive velocity at which 1RM was theoretically achievable [14]. Additionally, the coefficient of x (a) and y-intercept (b) were individualized for each participant. To establish a and b in the linear equation for each participant, scatter plots were produced, and a linear regression line was added using Microsoft Excel (version 18.12, Microsoft, Redmond, Washington, USA).

#### 2.3.2. Swimming Performances Tests

After a standard 800 m warm-up (600 m aerobic swim + 200 m progressive sprint), the 25 and 50 m front crawl tests were performed with a diving start. Five minutes of rest were integrated between the two tests. Thirty minutes after the 50 m front crawl test, the 25 and 50 m front crawl tests with arms only were performed with a water start. A pull buoy and an elastic between the two ankles were used to eliminate the effect of the legs during swimming. Five minutes of rest were performed between the two tests. All swimming performances were timed in seconds, as recorded by two timekeeping specialists with a stopwatches (SEIKO S120-4030, Tokyo, Japan) and were performed in a 50 m indoor pool with 27.2 and 25.9 °C water and air temperatures, respectively, and 64% relative humidity at 10.00 AM. All participants were informed, as in the dry land test, to eat their breakfast 3 h before the start of the test, and to avoid all other nutrition, including caffeine.

#### 2.3.3. Biomechanical Variable Tests

To eliminate the start and end effects of the race, only a distance of 10 m (between the 7.5 and 17.5 m marks) was evaluated for the kinematical variables of the 50 m front crawl. A surface video camera, Sony SNC VB 603 (50 Hz, full HD, 1080 p), was used to record the kinematical variables. The camera was placed about 5 m above the water and about 10 m away from the swimming line, laterally to the 10 m measuring zone. In addition, Kinovea software version 0.8.15 (Joan Charmant & Contrib., kinovea.org) was used to analyze the video sequences. The velocity was determined from the time taken to cover the 10 m. The stroke rate (SR) was assessed from the time taken to complete three consecutive stroke cycles. In addition, the stroke length (SL) was calculated from the ratio between the velocity and the corresponding stroke rate [15]. The stroke index (SI) was computed by multiplying velocity by stroke length [16,17].

### 2.4. Statistical Analyses

SPSS 26.0 (SPSS Inc., Chicago, IL, USA) was used for statistical analysis. All data are presented as mean and standard deviation. Normality was assessed for all variables using the Kolmogorov–Smirnov test, and all variables were normally distributed (*p* > 0.05). Linear regression analysis was used to predict the 1RM push-up performance. In addition, relationships between the 1RM push-up and swimming performance were determined by the Pearson product–moment correlation. Therefore, the correlation coefficients were interpreted as: small (0.1 to 0.3), moderate (0.3 to 0.5), large (0.5 to 0.7), very large (0.7 to 0.9), and nearly perfect (0.9 to 1.0) [18].

## 3. Results

**Figure 2:**
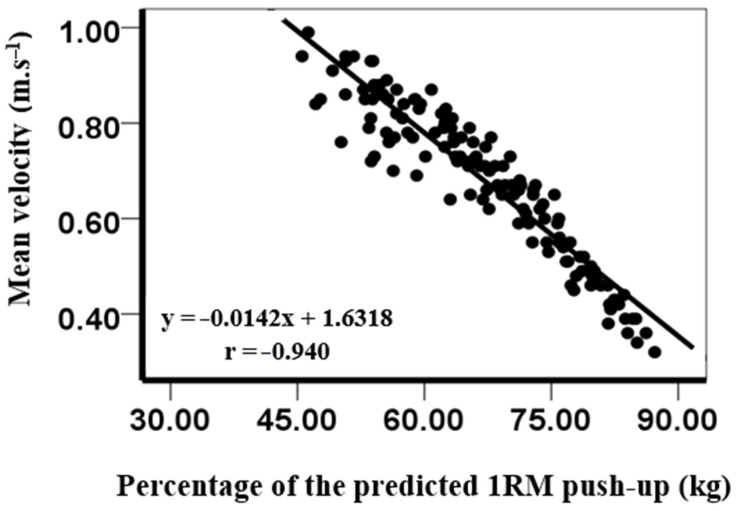
Relationship between mean velocity at each load and the percentage of the 1RM push-up.

Our results showed that the predicted 1RM push-up was 82.98 ± 9.95 kg. However, the load lifted during push-up conditions was between 44.69 ± 7.55 kg for the own body mass condition and 66.67 ± 8.66 kg for the + 30 kg condition. For instance, the percentage of body mass (+ weight vest) that had to be lifted during the push-up conditions was from 61.0 ± 3.47% to 64.6 ± 3.1%. The mean velocities during the lifts with different loads in the push-up were from 0.85 ± 0.07 m∙s^−1^ for the own body mass condition to 0.46 ± 0.07 m∙s^−1^ for the + 30 kg condition. A significant nearly perfect correlation was observed between the mean velocity at each load for each participant and the percentage of 1RM in the push-up (r = −0.94, Table 2 and Figure 2).

**Figure 3:**
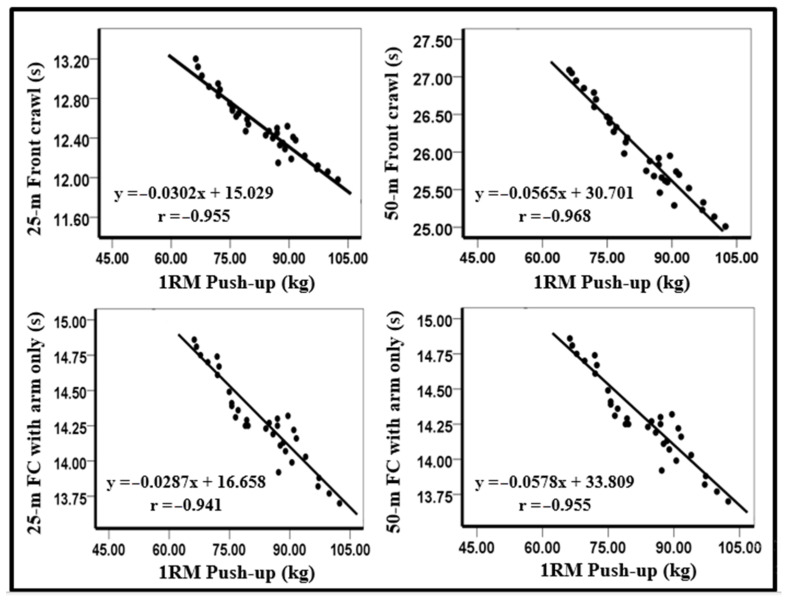
Relationship between swimming performance and the calculated predicted 1-RM push-up. FC: front crawl.

The results of the present study revealed a significant nearly perfect correlation between the calculated predicted 1RM push-up and the 25 (r = −0.96) and 50 m front crawl (r = −0.97). Our results also indicated a significant nearly perfect correlation between the 1RM push-up and 25 (r = −0.94) and 50 m (r = −0.96) front crawl with arms only (Figure 3).

**Figure 4:**
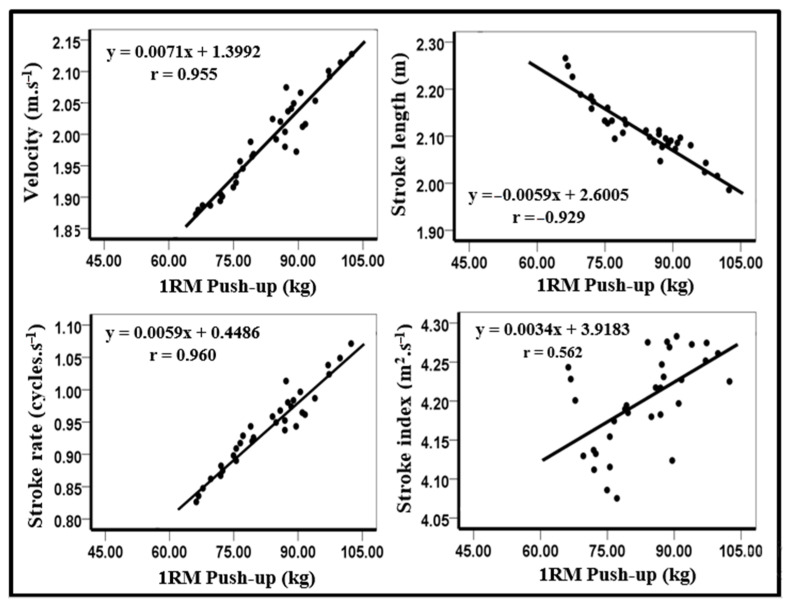
Relationship between kinematical variables and the calculated predicted 1-RM push-up.

Furthermore, significant nearly perfect correlations were observed between the 1RM push-up and the kinematical variables velocity (r = 0.96), stroke length (r = −0.93), and stroke rate (r = 0.96) in the 50 m front crawl, while a large significant relationship was found between the 1RM push-up and stroke index (r = 0.562, Figure 4).

## 4. Discussion

This study aimed to investigate the correlation between the calculated predicted 1RM push-up based upon the load–velocity relationship, swimming performance, and kinematical variables in the 50 m front crawl in competitive swimmers.

Our results showed a nearly perfect association between the 1RM push-up, all front crawl swimming performances and most kinematical variables during the 50 m crawl. Since no study has investigated the relationship between push-up performance and swimming performance, it is difficult to compare the findings with other studies. However, Keiner et al. [4] studied the correlation between the maximum upper and lower body strength and swimming performance (50 and 100 m front crawl) and found large to very large correlations with the 1RM bench press and back squat (r = 0.79, r = 0.67, respectively) in moderately trained male swimmers (age = 17.5 ± 1.6 years). In young male water polo players (age = 11.9 ± 1.3 years), Keiner et al. [3] showed a significant correlation between the 15 and 25 m front crawl and the 1RM bench press and arm span (r = 0.71, r = 0.72, respectively). As van den Tillaar and Ball. [8] showed that there is a near-perfect correlation between maximal bench press performance and push-ups, it is possible to compare these previous studies using the bench press with the present study. Therefore, testing upper body strength measured with push-ups seems to be a good predictor for front crawl swimming performance in competitive swimmers.

Furthermore, our results also showed a nearly perfect correlation between the 1RM push-up and 25 and 50 m swimming performance with arms only. For, instance Morouço et al. [19] showed that swimming with arms only accounts for 70.3% of the estimated relative contribution in the 50 m front crawl. The same authors [19] showed that to improve the 50 m velocity, swimmers are highly dependent on the maximum forces they can perform with arms only (r = 0.77). For this, it is very important to increase the maximum upper body strength to improve force propulsion in the front crawl with arms only and, consequently, to optimize the swimming performance [19].

Moreover, the predicted 1RM push-up showed very large to nearly perfect relationships with the kinematical variables, which was not surprising. However, the velocity was also calculated by multiplying the stroke rate by the stroke length (V = SR × SL). In addition, an increase in SR and/or SL increased the velocity [20]. However, short swimming distances are characterized by high velocity and maximum upper body strength [4,20,21]. Earlier studies only investigated the kinematical variables in relation to muscle strength by using the torque measured during isometric and isokinetic measurements of the upper body. Gola et al. [22] also found a significant correlation between the 25 and 50 m swimming velocity in the front crawl and the relative sum of the total torque for the upper extremity muscles (r = 0.60, r = 0.57, respectively) in university students with 2 years of mean competitive swimming experience. However, the difference in the years of experience in resistance training among the university students [22] and the competitive swimmers participating in this study may explain the discrepancy in the results obtained with this earlier study.

That such a high correlation was found between the maximal push-up performance and front crawl swimming performance over short distances and its kinematical variables is not surprising, since most of the muscles used during the push-up, i.e.: the shoulder muscles (rotator cuff, trapezius, deltoid) and arm muscles (biceps brachii and triceps brachii), are among the main muscles active during the front crawl [23].

During the push-up, around 61 to 65% of body mass (no extra load-30 kg weight vest respectively) were the loads lifted, which is in accordance with the findings of van den Tillaar and Ball. [8]. This is important information when planning to evaluate push-up performance based upon the load–velocity relationship when you have no access to a force plate to measure the forces. Furthermore, this information is also useful when using push-ups with extra loads (e.g., a weight vest) in planning training.

On the other hand, maximum upper body strength and swimming performance were improved by training programs that included the bench press (BP) and medicine ball throw (MBT) exercises [1,15]. Amara et al. [15] showed that nine weeks of dry land training with BP and MBT can improve the performance of 1RM BP (12.11 ± 1.79%) and sprint swimming performances of the 50 m front crawl (4.22 ± 0.18%) in male competitive swimmers (age = 16.5 ± 0.30 years). In the same context, Lopes et al. [1] revealed that eight weeks of dry-land training including BP and MBT can improve the 1RM BP (14.92%) and the performance of 50 m front crawl (3.98%) in university swimmers of the national level (age = 20.55 ± 1.76 years). According to our results, nearly perfect relationships were found between the 1RM push-up and swimming performance. For this reason, push-up exercises can be also included in the future studies that verify the effect of dry land training on swimming performance.

This study has some methodological limitations that warrant discussion. First, the participants may not have much experience with push-ups with weighted vests of different loads, which could influence the load–velocity relationship and, therefore, the predicted 1RM. In addition, the actual 1RM push-up was not been established, so it was not possible to make a comparison of the predicted 1RM to the actual 1RM. In a future study, an upper body training intervention is suggested, in which push-up performance is tested to investigate if swimming performance increases as much as push-up performance. In addition, in future studies it is necessary to examine the relationship between 1RM push-up prediction and swimming performance in female swimmers, and in swimmers of other training levels and other age categories (e.g., pre-pubertal or senior swimmers). On the other hand, the testing of swimming performance using a stopwatch represents a limitation in terms of test accuracy. Future studies should use electronic timing systems (e.g., Omega system).

## 5. Conclusions

The outcomes of this study revealed nearly perfect relationships between the 1RM push-up, swimming performance, and kinematical variables (velocity, stroke rate, stroke length, and stroke index) in the front crawl. This method is a low cost and time-effective alternative. For this reason, strength and conditioning coaches and swimmers could utilize the push-up exercise to predict maximum upper body strength to optimize swimming performance in short races.

## Figures and Tables

**Table 1:** Physical experience and anthropometric characteristics of the swimmers (mean ± SD).

Swimmers Characteristics	National Competitive Male Swimmers (N = 33)
Age (yr-old)	16.46 ± 0.59
Height (cm)	180.56 ± 5.69
Body mass (kg)	72.82 ± 8.41
Swimming training experience (years)	9.50 ± 0.71
Resistance training experience (years)	6.08 ± 0.37

**Table 2:** Mean load and velocity in the four push-up conditions.

Load	1	2	3	4
Push-up conditions	0 kg	+10 kg	+20 kg	+30 kg
Body mass plus weight vest (kg)	72.82 ± 8.41	82.82 ± 8.41	92.82 ± 8.41	102.82 ± 8.41
Actual load lifted (kg)	44.69 ± 7.55	52.20 ± 8.12	59.11 ± 8.31	66.67 ± 8.66
% of body mass (+weight vest)	61.00 ± 3.37	62.69 ± 3.49	63.41 ± 3.18	64.60 ± 3.14
Mean velocity (m∙s^−1^)	0.85 ± 0.07	0.76 ± 0.07	0.65 ± 0.06	0.46 ± 0.07

## Data Availability

The data presented in this study are available on reasonable re-quest from the corresponding author.
